# Impact of Demographic, Socioeconomic, and Psychological Factors on Glycemic Self-Management in Adults with Type 2 Diabetes Mellitus

**DOI:** 10.3389/fpubh.2016.00195

**Published:** 2016-09-12

**Authors:** Alicia A. Gonzalez-Zacarias, Ana Mavarez-Martinez, Carlos E. Arias-Morales, Nicoleta Stoicea, Barbara Rogers

**Affiliations:** ^1^Department of Anesthesiology, The Ohio State University Wexner Medical Center, Columbus, OH, USA

**Keywords:** diabetes mellitus, type 2 diabetes mellitus, social factors, glucose control, glycosylated hemoglobin

## Abstract

Diabetes mellitus (DM) is reported as one of the most complex chronic diseases worldwide. In the United States, Type 2 DM (T2DM) is the seventh leading cause of morbidity and mortality. Individuals with diabetes require lifelong personal care to reduce the possibility of developing long-term complications. A good knowledge of diabetes risk factors, including obesity, dyslipidemia, hypertension, family history of DM, and sedentary lifestyle, play an essential role in prevention and treatment. Also, sociodemographic, economic, psychological, and environmental factors are directly and indirectly associated with diabetes control and health outcomes. Our review intends to analyze the interaction between demographics, knowledge, environment, and other diabetes-related factors based on an extended literature search, and to provide insight for improving glycemic control and reducing the incidence of chronic complications.

## Introduction

Diabetes mellitus (DM) is frequently reported as one of the most common chronic diseases (1). According to the World Health Organization (WHO) global report on diabetes in 2016, an estimation of 422 million people worldwide were living with the disease by 2014, representing an increase of 3.8% since 1980 (2). In the United States, Type 2 DM (T2DM) is the seventh leading cause of morbidity and mortality (3). Individuals with diabetes require chronic management because of the high risk for long-term complications, such as coronary artery disease, end-stage renal disease, hypertension, stroke, retinopathy, neuropathy, and lower limb amputations (4).

**Figure 1:**
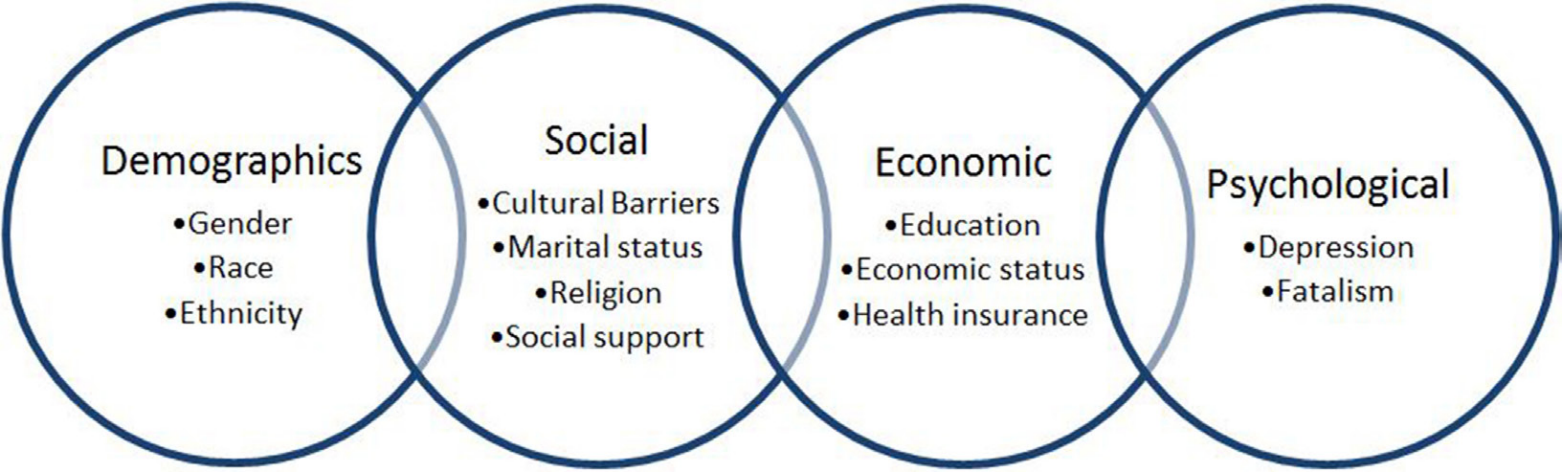
**Social factors influencing glycemic control in type 2 diabetes mellitus patients**.

For preventive purposes, identification of diabetes risk factors is of great importance. Well-defined diabetes risk factors include obesity, dyslipidemia, hypertension, family history of DM, and sedentary lifestyle. Equally important, but less precisely defined, are sociodemographic, psychological, and socioeconomic conditions, including gender, race, education, depression, and neighborhood environment, presumed to be associated with diabetes control and overall health outcomes (Figure 1) (4). For this reason and due to the high prevalence of T2DM worldwide, the need for identifying effective medical care with an adaptable disease self-management is fundamental. Achieving an optimum glycosylated hemoglobin (HbA1c) level demonstrates control of the disease and, therefore, prevention of its complications (3).

The purpose of this literature review is to examine current understanding of the social determinants affecting diabetes care in the United States, and to make recommendations for future research.

## Demographic Factors

Type 2 diabetes mellitus is a chronic disease affecting 9.7 million women and 10.9 million men among middle age and older adults in United States (5, 6). Analyzed by racial and ethnic groups, the prevalence is 13.1 million non-Hispanic whites, 3.2 million non-Hispanic blacks, 2.5 million Hispanic/Latino Americans, and 117,994 American Indians Natives, with the Black and Hispanic racial groups having the highest risk of complications and mortality (5).

Uncontrolled diabetes can lead to poor health status and has been associated with disability, micro and macrovascular complications, and premature death (7). Preventing complications and minimizing the risk of early mortality require modifications of lifestyle behaviors (6, 8). This regimen involves adherence to medication, improved diet, active lifestyle, self-monitoring blood glucose level, and good communication with health-care professionals. Glycemic control is likely to differ according to gender, ethnicity, culture, or marital status (8).

### Gender

Gender differences in glycemic control have been reported, although the findings have been mixed. Some studies have demonstrated that women are more likely to have better glycemic control compared to men, whereas other studies have shown the opposite (6). For instance, in a retrospective study including 263 patients, Roy et al. determined metabolic and behavioral factors associated with optimal glycemic control. Among all patients, 53.6% were males and 46.4% were females; 66.4% males were associated with suboptimal T2DM control, reporting a mean HbA1c of 8.87%. On the contrary, from the total of female gender, 56.9% had an optimal control with a mean HbA1c of 6.24%. Also, male gender was associated with higher incidence of other comorbidities, such as congestive heart failure and peripheral arterial disease (9).

Moreover, Mondesir et al. examined the association between illness-related diabetes social support (IRDSS) and glycemic control. This cross-sectional study included 914 middle age and older diabetic patients who completed a health and retirement survey. HbA1c levels were obtained to measure good glycemic control, considering <8.0% as normal. The prevalence of good glycemic control was 48.9% among women and 51.1% among men. Men reported higher levels of social support for following a meal plan and for taking care of their feet when compared to women. IRDSS has shown to be an important barrier for women with poor diabetes self-management (6). In another study, Beverly et al. explored the role of spousal support in diet-related diabetes management using data collected from 30 middle-aged and older married couples. The authors showed that spousal control over food preparation was relevant to men diagnosed with diabetes. Husbands who relied on their spouses to maintain a healthful diet exhibited low self-control. Women diagnosed with diabetes exhibited higher self-control despite perceived lack of support from their husbands. Committed spousal support was significant when encouraging patients with diabetes to adopt more healthful eating patterns (10). These findings may be explained in part by the operational definitions of social support, the variety of scales, and differences in the conceptualization and perception of social support by each individual.

Similarly, Mansyur et al. explored the extent of perceived social support and barriers associated with self-efficacy and self-care diabetes adherence. The study recruited 248 Hispanic men and women with uncontrolled T2DM. Participants were randomized to receive a culturally targeted intervention for diabetes management. Compared to men, women were less likely to receive support, faced more barriers, had less self-efficacy, and had lower levels of diabetes self-management leading to poor glycemic control. This may be related to cultural and social norms, such as gender role and the importance of family. For Hispanic women, prioritizing relatives’ needs over their own is typically reported (11).

### Race and Ethnicity

Studies have demonstrated the relationship between ethnicity and self-care behavior among T2DM individuals. In a cross-sectional study, Thackeray et al. showed that diabetes management significantly varied among ethnic groups. The authors categorized ethnic groups as non-Hispanic Whites, non-Hispanic Blacks, Hispanics, Asians, American Indians, and others. The study recruited 11,217 individuals with DM. Hispanics were significantly less likely to be educated on how to manage their diabetes and less motivated to check their blood glucose, HbA1c, and cholesterol levels. This group was characterized by poor feet self-examination and fewer dilated eye exam visits. The study showed that Hispanics, Blacks, and Asians, preferred to take oral hypoglycemic medications rather than subcutaneous insulin to control diabetes. Overall, white patients scheduled regular visits for dilated eye exam and were found to have better glycemic control based on HbA1c and cholesterol levels (12).

A survey study published by Correa-de-Araujo et al. investigated gender differences across ethnic groups in 2,365 T2DM individuals. The study showed no significant difference between HbA1c and lipid profiles measured in both genders from all ethnic groups. Furthermore, the rate of hospital admissions for uncontrolled diabetes was 25 per 100,000 for women and 27 per 100,000 for men. Despite those findings, the hospitalization rate was found to be higher in non-Hispanic Blacks and Hispanics (5).

A retrospective study by Heidemann et al. that included 25,123 diabetic patients aimed to determine race as an independent risk factor for controlling glycemic index after adjusting for socioeconomic status (SES). The authors identified race as an independent variable in diabetes control among patients with socioeconomic stability (13).

Reallocating resources to improve programs for diabetes self-care will promote better access to health care for different racial/ethnic groups, especially for Black men and Hispanic women (5, 12). Self-management education programs should be tailored to lifestyles, gender, and race in order to prevent complications and decrease the incidence of premature deaths in T2DM individuals (5).

## Social Factors

### Cultural Barriers

Key components of diabetes self-management education include regular glucose monitoring, healthy nutrition, and physical activity. Unfortunately, immigrants face barriers to effective diabetes control due to the predominance of low income and education levels, language barriers, cultural beliefs, and limited social and medical support (14).

Language barrier was identified as the most important factor influencing lack of patient–physician communication, contributing to poor diabetes knowledge, and self-management (14). In the United States, Hispanic and Asian patients reported poor glycemic control and worse health outcomes (15–17). Due to mutual language-concordant care provided by foreign-trained physicians, limited English proficiency became an independent factor in glycemic control data analysis among immigrants (15, 18).

Smith-Miller et al. explored T2DM individuals’ self-management among 30 Hispanic immigrants with limited English proficiency. The majority of the participants were Mexican females with low educational achievement. Based on data analysis, 59% of the participants were obese with HbA1c values above the intended target cut off (<7%). Low education and diabetes knowledge were found to be important limiting factors for achieving HbA1c <7%. The study concluded that the majority of participants did not effectively manage their disease (17).

Baig et al. examined the association between English language ability and glycemic control in 167 Hispanics with T2DM. Overall, 38% of the participants reported speaking English very well, 21% well, 26% not very well, and 14% not speaking English at all. Patients who spoke English very well were younger compared with all other groups. Unexpectedly, the authors found that English ability had a *U*-shaped relationship with glycemic control. The HbA1c was higher (8.0 ± 1.9 and 8.6 ± 1.0) in Hispanics who spoke English very well and those who did not speak English at all, compared with Hispanics who spoke English well (6.0 ± 1.1) (15). These results may be explained by acculturation to mainstream American dietary practices by patients who speak English very well; Hispanics who speak well or not at all are more tied to their habitual Latin–American diet low in fat and high in fiber with a better glycemic control. However, these findings may contradict with Carbone et al. study (19) who reported that Latin–American diet is also high in fats, carbohydrates, and sugars, thus conveying patients to a poor glycemic control.

Dietary management is difficult to achieve for the majority of patients, especially female patients, considering traditional family meal preparation habits. Knowledge of nutrition-related cultural variations among different ethnic populations is essential in order to provide appropriate advice to prevent and treat T2DM based on modification of traditional diets (20). This is especially true because Hispanic diet tends to be high in sugars and carbohydrates. By the same token, physical activity is limited mainly due to the topographical location where these populations reside, where crime and violence is usually predominant (14, 19).

### Marital Status

Diabetes care regimen is influenced by marital status, as self-management often needs the participation of a partner or spouse to better achieve optimal glycemic control. Studies have shown that a partner’s participation in diabetes education programs improves outcomes when compared to patients with no partner support. Results of these studies demonstrated poor glucose control and more complications in the latter group (10, 21).

Trief et al. studied 78 diabetic patients on insulin therapy who were married for at least 1 year. The authors demonstrated a relationship existing between partner’s support and diabetic outcomes, with more support corresponding to less distress in individuals with diabetes. This study supports the recommendation of involving the partner to maximize quality of diabetes management (21).

### Religion

Diabetes self-management is strongly influenced by cultural and spiritual beliefs. DM patients with religious beliefs have lower depressive symptoms than those with none, possibly due to the healing themes, emotional support, and practical assistance that religion may provide (22, 23). Patel et al. demonstrated on a group of 67 DM patients that fatalistic attitudes influence self-management practices. Some participants attributed the onset of their diabetes to fatalism or being ordained by God (24).

### Social Support

Interventions to improve glycemic control should not target exclusively diabetic patients. Family members need to expand their knowledge of the disease for proper care of their relatives. Considering more than a quarter of adults aged 65 and older have diabetes, the support of family members plays a strong role in accomplishing an accurate treatment (25). A group of patients’ who participated in a diabetes intervention program along with their families improved their diabetes knowledge. The mean HbA1c was 8.5% at the beginning of the first session into the program and 7.7% at the completion of it (26).

Functional support in the form of engaging in recreational and physical activities with family and friends may be an effective intervention strategy to promote self-care behaviors in T2DM patients and weight loss (27). Thus, receiving group care is associated with higher glycemic control and reduced fatalism (28).

## Economic Factors

Type 2 diabetes mellitus has been reported to be predominant in developing countries when compared with developed countries; these differences in health outcome between nations can be attributed to disparities in educational program systems and economic development (1, 29, 30).

The level of education and income among immigrant patients with diabetes may contribute to the difficulties in understanding and managing their disease. High cost of medications and disease-specific care supplies represents a limitation for the proper management of diabetes in this population. Moreover, the Hispanic productive patient population faces medication adherence problems due to work commitments.

Previous studies have directly and indirectly linked low SES to poor health outcomes (31). The stress of the economic inequality can increase the risk of poor glucose control and diabetes complications through the inability to purchase healthy food, to participate in exercise or recreational activities, to manage capillary glucose at home, and to access the health care system to receive proper treatment. Also, economic distress can trigger unhealthy weight gain, smoking, and heavy alcohol consumption, increasing the risk of developing chronic complications (31, 32).

### Education and Economic Status

Dupre et al. studied the educational differences in glycemic levels, and the factors contributing to the survival differences in older adults with diabetes. High school or greater education was associated with better glycemic control and higher survival rates at follow-up when compared to those with lower education (3, 33).

Similarly, Walker et al. investigated the independent effects of socioeconomic and psychosocial determinants of health on diabetes knowledge, self-care, and quality of life. They demonstrated that diabetes knowledge was associated with college education and an income of >USD$20,000. Also, better diabetes outcomes were significantly associated with higher SES, self-efficacy, and quality of life (34).

### Health Insurance

The accessibility for health insurance remains suboptimal despite recent changes in health care reform (35). The lack of health insurance has been associated with poor health outcomes and reduced quality of life among adults who suffer from chronic diseases, particularly diabetes (35, 36).

Unfortunately, uninsured patients with limited financial resources are prone to fewer medical visits and experience difficulty obtaining diabetes testing supplies, medications, and access to healthy food, resulting in poor glycemic control and higher rate of hospitalizations (36, 37).

The cost of diabetes self-management plays an important role in achieving optimal metabolic control (38). Insurance coverage for diabetes-testing supplies varies across the nation and sometimes does not include supplies coverage. According to Bowker et al., patients without insurance confront more difficulties getting self-monitoring test strips, which results in poorer glycemic control than those with private insurance (39).

A retrospective study published by Bailey et al. examined the association between health insurance coverage and receipt of diabetes preventive care. The authors compared insured patients with uninsured and discontinuously insured patients. Uninsured patients experience poorer glycemic control as a result of significantly lower medical care visits (40).

However, a low-income and uninsured status is not always associated with poor glycemic control. Madden et al. compared strategies of uninsured patients who successfully managed their diabetes with those who unsuccessfully controlled it. From the total of 26 participants recruited, 17 were found to have successful diabetes control with a median HbA1c of 6.5% compared with 10.2% in the unsuccessfully managed group. The presence of a diabetic family member positively influenced diabetes management because they served as an observational learning model. Patients in the successfully managed group reported having more family members with diabetes, visiting their health care provider more frequently, and participating more actively in diabetes support groups. This role model prepared patients to understand their condition and helped them to formulate their own self-management behaviors (36).

Poor diabetes management generates high hospitalization costs, as shown by Fisher and Ma who studied the association between potentially preventable hospital admissions among T2DM patients with different health insurance. Health insurance was categorized as Medicaid, Medicare, uninsured, and private. The admission rate was 3.1 and 2.4 times higher in uninsured and Medicaid patients when compared with Medicare and privately insured. Uninsured and Medicaid patients may not be able to optimally seek preventive care services, increasing the incidence of acute complications requiring hospital admissions (37).

## Psychological Factors

Psychosocial variables, including depression, fatalism, religion, and social support, have been recognized as strong predictors of diabetes management (41).

### Depression

Diabetes mellitus is a chronic debilitating disease with physical and psychological complications. Depressive symptoms are often associated with worse diabetes control (42). Biologically, depression affects glycemic control *via* metabolically relevant pathways, including alterations in neuroendocrine and glucose metabolism. Negative mood, such as anger and sadness, results in unsuccessful adherence to self-management recommendations (43).

Patients with known diabetes have higher prevalence of depression compared to those with unknown diabetes (44). Almeida et al. demonstrated in a group of elderly men that the risk of developing depression progressively increased depending on the years that the patient becomes aware of the diagnosis (45). Parildar et al. applied a questionnaire to a group of 110 patients with T2DM. They found that 55.5% of the subjects were suffering from depressive symptoms. The symptoms were correlated positively with the duration of the diagnosis, being significantly higher among T2DM patients with an established disease when compared with newly diagnosed patients. However, there was no significant correlation between HbA1c levels and depression or coping strategies (46).

Receiving treatment for depression may improve glycemic control in patients with T2DM. A group of 196 diabetic patients with major depression were offered to receive treatment with fluoxetine. Only 43.95% of the subjects accepted to participate. After 8 weeks, fasting plasma glucose and HbA1c levels were found to be significantly lower in patients who accepted to receive treatment compared to those who denied treatment (47). Additionally, Czech et al. demonstrated that depressive patients with sleeping problems had significantly higher HbA1c levels (42).

### Fatalism

Diabetes fatalism is defined as “a complex psychological cycle characterized by perceptions of despair, hopelessness, and powerlessness” (48). The 12-item Diabetes Fatalism Scale (DFS-12) is a brief, easy to administer tool to measure emotional distress, religious or spirituality coping, and perceived self-efficacy. In DFS-12, a higher score represents greater diabetes fatalism. To construct the validity of DFS-12, the assessment was pilot tested on 20 adults with diabetes, and then administered to 216 primary care patients with T2DM. The DFS-12 significantly correlated lower mental health component scores with higher HbA1c levels (49).

Many factors are involved to achieve an effective diabetes self-management. Besides disease-related knowledge, other factors are influencing the decision to follow self-care regimen. Walker et al. completed a study of 378 subjects with T2DM to examine diabetes fatalism using DFS-12. The authors found that diabetes fatalism was significantly associated with poor medication adherence but was not significantly correlated with diabetes knowledge. The association between diabetes fatalism with other diabetes factors did not change in patients diagnosed with depression. Diabetes fatalism is more similar to a personality feature rather than a psychological symptom (48).

The influence of ethnicity on fatalism is another factor to correlate with diabetes self-management behavior. Seven focus groups were conducted on 39 African-Americans with T2DM. Most participants perceived diabetes as an inherited disease that they had no control over. Fatalism was associated with diabetes self-management and was multidimensional in this population (50). Nevertheless, Pijl et al. studied a group of individuals with family history of DM. Subjects who received hereditary risk information perceived it as the most important cause of diabetes. Furthermore, they reported healthy eating 3 months after receiving the information (51). Thus, this study demonstrated that communicating the familial risk of developing diabetes can increase personal control of the disease and reduce fatalism.

## Neighborhood Environment

Smalls et al. evaluated the relationship between neighborhood characteristics, self-care behavior, and glycemic control through a questionnaire survey. This study found that self-care behaviors and neighborhood esthetics had direct effects on glycemic control. Social support and access to healthy foods had a direct effect on self-care, and social support had an indirect effect on glycemic control *via* self-care behavior. This study elucidated that esthetic neighborhoods directly inspire individuals to improve their high glucose level through nutritional selections and increased physical activity (52).

Likewise, de Vries McClintock et al. examined whether neighborhood social environment was associated with medication adherence and glycemic control among T2DM individuals. The authors found that residents living in neighborhoods with high social affluence and high residential stability were more likely to have an adherent pattern. These results confirm that the neighborhood environment is an important factor for the adherence to the treatment and subsequent outcomes (53).

Additionally, living in a neighborhood with limited access to healthy food and safe places to exercise can be a significant barrier for diabetes self-care and poorer glycemic control (14, 54). O’Donnell et al. suggested that patients living in a neighborhood with high social influence, high residential stability, and high neighborhood advantage are much less likely to experience depression (55).

Bodicoat et al. investigated the relationship between neighborhood greenspace with T2DM individuals in a large multiethnic population. The authors concluded that the increase of neighborhood greenspace was associated with significantly lower levels of screen-detected T2DM because of more health promoting behavior, particularly physical activity in better accessibility areas (56).

## Self-Management Education

Individuals diagnosed with T2DM require daily self-management, and the performance of complex care activities (57). Currently, around 50% of the population diagnosed with DM does not achieve the recommended target of the HbA1c level (<7.0%), blood pressure (<130/80 mmHg), and low-density lipoprotein cholesterol (<100 mg/dL) (58). Consequently, the American Diabetes Association (ADA) and the American Association of Clinical Endocrinologists recognize diabetes self-management education (DSME) and diabetes self-management support (DSMS) as a part of the diabetes control (57, 58). DSME is the process of facilitating the knowledge, skills and abilities for diabetes self-care, and DSMS denotes the support for implementing coping skills and behaviors needed for self-management (57).

A systematic review published by Chrvala et al. compared the impact of receiving diabetes self-management education and not receiving education on HbA1c levels in adults diagnosed with T2DM. Patients who engaged in educational interventions achieved reduction of the HbA1c level from −0.1 to −2.50 compared with those who were not intervened. The authors found that diabetes self-management education was associated with significant improvement of glycemic control (58). Self-management knowledge has a positive impact of on the reduction of HbA1c levels and decreases the progression of the disease (59).

These studies support that educational intervention is a favorable strategy for better clinical outcomes. Nonetheless, African-American’s are the second highest burden population with diabetes in the United States, this model has not been extensively reported in this ethnic group. Tang et al. demonstrated that, besides receiving a diabetes educational intervention, 106 African-American did not show any change in HbA1c levels at 3- or 12-month follow-up (60).

## Conclusion

While a patient’s knowledge is important, it is unlikely that this alone would profoundly improve diabetes control. Due to the enormous public health burden of T2DM, future work should describe aspects associated with meeting glycemic control recommendations using a multifaceted approach. New strategies, such as adding more diabetes educational programs to the treatment plan, increasing community physical and recreational activities, providing more access to health care providers, etc., should be implemented to reduce risk factors in order to achieve better health outcomes for these patients. Understanding the interaction between demographics, knowledge, environment, and other diabetes-related factors may provide insight for improving glycemic control and reducing the incidence of chronic complications.

## Author Contributions

All authors listed, have made substantial, direct and intellectual contribution to the work, and approved it for publication.

## Conflict of Interest Statement

The authors declare that the research was conducted in the absence of any commercial or financial relationships that could be construed as a potential conflict of interest.
